# Does the oxytocin receptor polymorphism (rs2254298) confer 'vulnerability' for psychopathology or 'differential susceptibility'? insights from evolution

**DOI:** 10.1186/1741-7015-10-38

**Published:** 2012-04-17

**Authors:** Martin Brüne

**Affiliations:** 1 Research Department of Cognitive Neuropsychiatry and Psychiatric Preventive Medicine, Department of Psychiatry, Psychotherapy and Preventive Medicine, University of Bochum, LWL University Hospital, Alexandrinenstrasse 1, D-44791 Bochum, Germany

## Abstract

The diathesis-stress model of psychiatric conditions has recently been challenged by the view that it might be more accurate to speak of 'differential susceptibility' or 'plasticity' genes, rather than one-sidedly focusing on individual vulnerability. That is, the same allelic variation that predisposes to a psychiatric disorder if associated with (developmentally early) environmental adversity may lead to a better-than-average functional outcome in the same domain under thriving (or favourable) environmental conditions. Studies of polymorphic variations of the serotonin transporter gene, the monoamino-oxidase-inhibitor A coding gene or the dopamine D4 receptor gene indicate that the early environment plays a crucial role in the development of favourable versus unfavourable outcomes. Current evidence is limited, however, to establishing a link between genetic variation and behavioural phenotypes. In contrast, little is known about how plasticity may be expressed at the neuroanatomical level as a 'hard-wired' correlate of observable behaviour. The present review article seeks to further strengthen the argument in favour of the differential susceptibility theory by incorporating findings from behavioural and neuroanatomical studies in relation to genetic variation of the oxytocin receptor gene. It is suggested that polymorphic variation at the oxytocin receptor gene (rs2254298) is associated with sociability, amygdala volume and differential risk for psychiatric conditions including autism, depression and anxiety disorder, depending on the quality of early environmental experiences. Seeing genetic variation at the core of developmental plasticity can explain, in contrast to the diathesis-stress perspective, why evolution by natural selection has maintained such 'risk' alleles in the gene pool of a population.

Please see related manuscript: http://www.biomedcentral.com/1741-7015/10/37

## Background

The idea that individuals are at elevated risk of developing a psychopathological condition due to their genetic make-up, particularly when exposed to adverse environmental conditions, known as the diathesis-stress model [1], has become the prevailing theoretical concept in psychiatry. The model further entails that people who do not carry 'vulnerability genes' are less susceptible to adversity. One of the most widely cited examples of gene-environment-interaction was published by Caspi *et al*. [2] who demonstrated that persons carrying the low-activity variant of the monoamine oxidase A (MAO-A) enzyme are more likely to develop antisocial personality disorder under adverse environmental conditions during childhood compared to individuals endowed with the high-activity allele. Put differently, subjects who degrade biogenic amines like dopamine and noradrenaline slowly are more vulnerable to develop, as children, attention-deficit hyperactivity disorder (ADHD) and conduct disorder, and, as adults, antisocial personality disorder when having suffered early adversity such as maltreatment or abuse. Conversely, it has been argued that the absence of adversity has the potential to compensate the genetic vulnerability, also known as 'maternal buffering' [3]. Other examples of gene-environment interaction include the s-allele of the serotonin transporter gene (5-HTTLPR), which predisposes to depression if accompanied by stressful life-events [4], and the seven-repeat variant of the dopamine receptor D4 gene (DRD4), which increases the vulnerability for externalizing problems and ADHD in children whose mothers are insensitive to their children's needs [5].

While it is indisputable that this line of research has greatly advanced our understanding of the role of gene-environment interaction in psychopathology, the diathesis-stress model, as currently conceived, falls short of explaining why many of these vulnerability genes have undergone recent positive selection in human evolution, because it is implausible to assume that natural selection has favoured allelic variants only to increase one's vulnerability to adversity. In other words, there must be a flipside of the coin.

Evidence from cross-cultural genetic studies suggests, for example, that the seven-repeat variant of the DRD4 gene emerged some 50,000 years ago in human populations, whereas the four-repeat variant is the ancestral version of this gene [6]. The seven-repeat allele has repetitively (though inconsistently) been associated with the personality trait 'novelty seeking', a human attribute that arguably may have conferred a reproductive advantage in recent human history [7,8]. Similarly, Lesch and colleagues have shown that, while carrying the s-allele of the HTTLPR predisposes to depression if associated with adverse events, the same variation is linked to superior cognitive performance in several domains and increases social conformity [9,10]. The latter example is prototypical for a 'balanced polymorphism', that is, a single nucleotide polymorphism (SNP) may exert disadvantageous effects in one domain, which are compensated by advantageous effects in another domain. A balanced polymorphism also explains the frequency of a particular SNP in the general population, and why it has not been selected against.

Balanced polymorphisms are, however, implausible to account for those cases in which the compensatory effect happens to occur in the same domain. Instead, Belsky [11] has argued from an evolutionary point of view that a particular genetic variation that predisposes to pathology if associated with early adversity can have beneficial effects when environmental contingencies are developmentally more positive. To this end, Belsky *et al*. [12] have highlighted a methodological problem that is notoriously prevalent in most gene-environment-interaction studies. They argued that 'simply treating the absence of adversity as the "good" end of the environmental-exposure continuum and/or absence of a disorder as the "good" end of the psychological functioning continuum may lead to the under-detection of *differential susceptibility *[this author's emphasis] findings and an over representation of vulnerability ones.' Accordingly, they proposed that it is necessary to inquire whether the same genes that convey increased vulnerability to psychopathology under adverse environmental conditions have the potential to act in advantageous ways on psychological functioning when the environment is supportive or enriched [12]. Put another way, it may be more accurate to speak of differential susceptibility or plasticity conferred by genetic variation - that is, responsivity to both positive and negative conditions - rather than focusing one-sidedly on vulnerability [13].

In support of this ground-breaking and novel conceptualization, it has been demonstrated that the low-activity MAO-A variant is associated with lower than average prevalence of antisocial personality when children grow up in supportive environments [14]. Likewise, the s-allele of the 5-HTTLPR confers a lower risk for depression under favourable environmental conditions [15], and children carrying the seven-repeat variant of the DRD4 gene develop ADHD and externalizing problems less than average if their mothers behave in responsive ways to their children's needs [5].

The potential impact of this new perspective on gene-environment interaction and behavioural consequences, as far as psychopathological conditions are concerned, can hardly be over-estimated. At present, this approach has only been linked to the behavioural phenotype and not to the neuroanatomical correlates on which putative plasticity genes can also operate. If it is possible to demonstrate, however, that plasticity genes convey differential effects on both behaviour and matching neuroanatomical structures, the model would support the claim that the diathesis-stress concept should be refined in the direction outlined above.

Accordingly, this article seeks to explore the differential impact of genetic variation on behaviour and underlying anatomical structures, exemplified by a polymorphic variation (rs2254298) of the oxytocin receptor gene (*OXTR*). This SNP of the *OXTR *was chosen for several reasons. First, research on human social behaviour suggests that natural selection has favoured the processing of social information in primates, including humans, which culminates in the detection of intentions and dispositions of conspecifics (members of the same species). This key concept of human brain evolution, referred to as the 'social brain' hypothesis [16], proposes that a major driving force that ultimately led to the evolution of large hominid brains was the increasing complexity of ancestral social environments [17]. Oxytocin (OXT) is an evolutionarily conserved hormone that is critically involved in regulating social behaviour such as social recognition, mating, attachment and caring for offspring in virtually all mammalian species [18]. In support of this notion, recent studies have discovered that the administration of OXT has the potential to increase social (re)-cognition in humans [19,20], and even to partially restore impaired social cognition in neuropsychiatric disorders [21,22]. Moreover, evidence is accumulating that polymorphic variation of the *OXTR *impacts on attachment-related behaviour and social cognition in humans [23,24].

Secondly, The SNP rs2254298 of *OXTR *has been studied considerably with regard to its impact on various psychological dimensions, such as affect and temperament. In addition, this SNP is the only one at *OXTR *for which information on the interaction between genetic variation, attachment and the quality of early childhood experiences is available. Moreover, the SNP rs2254298 has been studied as regards neuroanatomical correlates of social cognition, and has undergone recent evolutionary change. All of these factors render this SNP of *OXTR *unique to address the question as to whether or not it could represent a candidate for the differential susceptibility hypothesis.

This article is organized as follows: first, it summarizes the effects of OXT on social cognition and the role of OXT in psychopathological conditions. Second, it highlights the findings of studies that have specifically dealt with the SNP rs2254298 of *OXTR *as far as psychological dimensions, attachment and neuroanatomy are concerned. Third, the implications (as well as limitations) of these findings for the differential susceptibility hypothesis with regard to rs2254298 are discussed. The article concludes with some ideas for future directions and research.

### Oxytocin, social cognition and psychopathology

For decades, OXT was best known in medicine for its role in parturition and lactation. Only recently, OXT has been intensely investigated for its potential impact on psychological dimensions such as social attachment and cognition [19]. In addition, OXT has become a target of clinically motivated research, particularly concerning its relationship to vulnerability to disorder and treatment of psychiatric disorders [21,22]. Consequently, based on observations that intranasal application of supra-physiological dosages of OXT improves 'mindreading' abilities [25], fear recognition [26] and trust in healthy subjects [27,28], as well as studies demonstrating the role of OXT in establishing secure attachment and bonding [29,30], research on the effect of OXT on psychopathological parameters and cognition in adult psychiatric disorders has seen a recent resurgence after a long period of lying dormant.

It was shown in the early 1970s that OXT has the potential to reduce psychotic symptoms in schizophrenia [31]. More recently, Feifel *et al*. [32] as well as Pedersen *et al*. [33] demonstrated that supra-physiological dosages of intranasally administered OXT improves mindreading in schizophrenia and reduces positive symptoms when administered as an adjunct treatment to antipsychotic medication. An improvement of social behaviour has also been reported in individuals with autistic spectrum disorders [34]. Other studies have focused on alterations of OXT concentrations in the cerebrospinal fluid (CSF) and serum. An initial study found elevated CSF levels of OXT in patients with schizophrenia compared to healthy controls, which were higher in those patients receiving antipsychotic treatment [35], whereas a follow-up study could not confirm these results [36]. However, another study demonstrated reduced OXT serum levels in patients with schizophrenia, which additionally predicted their ability to correctly identify facial emotions [37]. In trust-related interpersonal interactions, healthy controls showed increased plasma levels of OXT, whereas this effect was absent in patients with schizophrenia [38]. Recently, higher OXT serum levels in patients with schizophrenia have been found to be associated with reduced symptom severity compared to patients with lower OXT serum levels [39].

In depression, serum OXT levels have been found to be reduced, which included patients with bipolar depression [40]. This reduction seems to correlate with symptom severity [41], although another study suggests otherwise [42]. Moreover, postmortem analyses indicate an increased number of OXT-expressing neurons in the paraventricular nucleus in depression, which may be associated with the activation of the hypothalamic-pituitary-adrenal axis [43].

In anxiety disorders, OXT attenuated conditioned fear responses and amygdala activity [44] to become similar to the effects observed in psychologically healthy individuals [45]. Somewhat inconsistent with these findings, OXT plasma concentrations in social anxiety disorder were positively correlated with symptom severity and dissatisfaction with social relationships [46].

In a similar vein, women at risk of developing postpartum depression had lower OXT plasma concentrations than women who had no increased risk for postpartum depression, and OXT serum levels in mid-gestation predicted postpartum depression symptoms [47]. OXT has also been found to interact with the stress axis (particularly cortisol) [48], and a stressful early life - for example, through emotional abuse - have been linked to reduced OXT levels in the CSF [49]. This finding may be particularly relevant for borderline personality disorder, because traumatization is prevalent in this disorder and leads to reduced stress-tolerance and, hence, problems in emotion regulation. A recent study reported that stress reactivity improved in patients with borderline personality disorder upon administration of OXT [50], which contrasts with another study showing that trust and cooperation decreased in patients with borderline personality disorder as a function of anxious-ambivalent attachment [51]. This is consistent with a recent study in healthy subjects, which combined 'compassion-focused imagery' with OXT administration. This study demonstrated that individuals higher in self-criticism and lower in self-reassurance, social safeness and attachment security benefitted less from OXT compared to securely attached participants who had a more positive self-evaluative style [52].

Taken together, OXT seems to be involved in various psychopathological conditions. However, the diversity of findings regarding plasma or CSF levels of OXT as well as effects of intranasal OXT suggests a role for gene-environment interactions that is as yet not fully understood. Accordingly, the next section explores potential links between a polymorphic variation of *OXTR *(rs2254298), social behaviour, attachment and anatomical correlates of social cognition.

### Polymorphic variation of the oxytocin receptor coding gene and vulnerability to disorder

Recent studies have addressed the genetic variation of different loci within *OXTR *and their potential role in psychopathological conditions (reviewed in [21]). The human OXTR is a peptide comprising 389 amino acids. *OXTR *is located on the short arm of chromosome 3 (3p25) and has three introns and four exons. About 30 SNPs have been localized in the *OXTR *region, most of which are located in the intron sections [53].

On a MEDLINE survey using 'oxytocin receptor' and 'polymorphism' as key words, 11 matches were retrieved for the SNP rs2254298. This SNP is situated in the third intron of *OXTR*. Comparative genetics suggest that a mutation replacing guanine (G) by adenine (A) has taken place at some point during human evolution. Since the A allele is absent in other primates, it can be concluded that G is ancestral to A. Population genetics indicate that the frequency of the A allele differs widely between ethnicities. For example, in samples of European ancestry the A allele is quite uncommon, with the vast majority of individuals being homozygous for the G allele. In Asian populations, the frequency of the A allele is much higher, and around just 40% to 50% are GG carriers [54]. This difference in allele frequency needs to be kept in mind when comparing studies in different populations.

Several studies have examined the link of allelic variation of rs2254298 with psychopathological conditions. For example, the A allele has been linked to autism in a Chinese [55] and in a Japanese sample [56], but not in Caucasians [57]. Instead, in the latter study the G allele was associated with a higher risk for autism, a finding that is consistent with the association of a five-locus haplotype containing the rs2254298 SNP with autism in an Israeli sample [58].

With regard to emotion regulation and affective disorders, a haplotype block of seven SNPs containing rs2254298G was associated with lower scores of depressive affect in a Japanese sample of non-clinical individuals [59]. That is, individuals carrying at least one A allele had higher scores of depressive affect as measured using a semi-structured interview to assess one's temperament (TEMPS-A). This finding is consistent, though not identical to, a study by Lucht *et al*. [60], who reported that carriers of rs2254298G had (at trend level) higher values of positive affect compared to A carriers, as examined using the Positive and Negative Affect Schedule, an instrument that, unlike the TEMPS-A, refers to one's current mood, rather than temperament as a personality trait. Conversely, Costa *et al*. [61] reported that, within a clinical sample of Italian patients with unipolar or bipolar depression, carriers who were homozygous for rs2254298G (as opposed to GA or AA carriers) were over-represented in the group of patients with unipolar, but not bipolar, depression. In addition, with respect to attachment factors, GG carriers rated relationships significantly more often as secondary on the Attachment Style Questionnaire than GA or AA carriers. This finding suggests that an avoidant attachment style is more common in a clinical sample of patients with unipolar depression carrying the G allele of the rs2254298 SNP [61]. Interestingly, a recent study showed that the attachment style in healthy children was differentially associated with the presence or absence of an A allele according to the children's ethnicity. That is, the presence of at least one A allele of the rs2254298 SNP was associated with a significantly higher rate of secure attachment than in GG carriers. However, this effect was restricted to non-Caucasian children, whereas no such effect was found in Caucasian children [62].

Since the aforementioned studies have not addressed gene-environment interactions directly, it is difficult to judge the extent to which one's experience of early relationships (as revealed by attachment style) may or may not interact with the development of clinical symptoms. Thompson *et al*. [63] have demonstrated, however, that such a link might exist with respect to rs2254298. In a sample of nonclinical adolescent girls of mixed ethnic background, aged between nine and fourteen years, they found that girls carrying the AG genotype reported higher levels of depressive symptoms, greater social anxiety and physical symptoms of anxiety when having experienced early adversity as compared to GG carriers (the AA genotype was too rare, and thus excluded from the analysis). In this study, early adversity was defined by a history of maternal depression, that is, mothers who had at least two depressive episodes during their daughters' lifetime, whereas no early adversity was defined by the absence of maternal depression. Put another way, there is some evidence to suggest that the presence of an A allele of the rs2254298 SNP may confer increased risk of developing symptoms of anxiety if associated with early adversity, but at present there is no clear evidence for lower than expected rates of anxiety or depression in individuals with an A allele under favourable early environmental conditions. However, a closer inspection of Figure 1 in that article [63] suggests that the presence of an A allele in the absence of early adversity was associated with lower levels of depressive symptoms and lower social anxiety scores as compared to GG genotype carriers who scored higher on depressive symptoms and social anxiety (but not separation anxiety and physical symptoms of anxiety), regardless of whether or not the GG carriers were exposed to early adversity. This suggests that the GG genotype carriers were less responsive to environmental contingencies as regards the development of depressive symptoms and social anxiety compared to the AG genotype carriers of the rs2254298 SNP.

Consistent with this (tentative) interpretation, two anatomical studies have revealed that carriers who possess at least one A allele of the rs2254298 SNP exhibit greater amygdalar volumes bilaterally compared to G carriers. That is, GG genotype carriers have the smallest amygdala volume, AA carriers the largest volume, with AG genotypes lying in between. This association seems to be quite robust, as it was found in two independent samples, one of which comprised over 200 adult Japanese patients [64], and the other comprising 51 adolescent Caucasian girls [65], although the first of the aforementioned analyses has been contested for possible methodological issues [66,67].

When viewing the A genotype of the rs2254298 SNP of *OXTR *merely as a 'risk allele' for psychopathology, foremost autism, depression or anxiety disorders, it may appear plausible that greater amygdala volumes represent the anatomical correlate of greater vulnerability to one of these psychopathological syndromes. An equally parsimonious explanation is, however, that people who possess an AA or AG genotype of rs2254298 are differentially susceptible to develop a mental disorder only if the genotype is associated with early adversity. Under favourable environmental conditions, carriers of the AG or AA genotype may in fact have a reduced risk of developing a psychiatric disorder. Greater amygdala volume is, by no means, exclusively linked to an enhanced risk for autism. On the contrary, a recent study reported that amygdala volume was positively correlated with the size and complexity of one's social network. Specifically, the number of people in one's social network, as well as the number of different groups these contacts belonged to, correlated significantly with the volume of the left (and slightly less the right) amygdala in younger and older psychologically healthy adults of both sexes [68]. Taken together, these findings support the view that diathesis-stress models of individual SNPs are often just one side of the coin [12].

## Discussion

The present article sought to explore the question whether a polymorphic variation of the OXTR coding gene could be associated with differential susceptibility to psychiatric disorders. The basic premise was, in line with Belsky's theory [11], that genetic variation of the OXTR that increase the vulnerability to psychopathology can exert beneficial effects in the same domain when environmental contingencies are developmentally more positive. In an extension of the findings of Belsky *et al*. supporting this idea [12], a second goal of this review was to examine if such differential susceptibility to disorder could be linked to neuroanatomical correlates of the behavioural phenotype.

Consistent with the hypothesis that genetic variation at *OXTR *is possibly associated with differential susceptibility, carriers of the AG or AA genotype of the rs2254298 SNP have been found to be more vulnerable to developing a psychiatric condition, including autism [55-57], depression and anxiety disorders [60,61,63], with profound differences depending on ethnic background. In addition, variation at this polymorphic site of *OXTR *seems to impact the development of secure attachment [62], again with differences according to ethnicity. None of the aforementioned studies has taken into consideration potential gene-environment interaction in terms of the effects of early adversity or the absence thereof. To date, only one study gives at least some clues how this could be conceptually relevant. Thompson *et al*. demonstrated that the A versus G polymorphism of the rs2254298 SNP is differentially associated with the presence or absence of early adverse experience [63]. In this study, carriers with the AG genotype had a greater risk of developing depressive symptoms and social anxiety than GG genotype carriers when exposed to adverse events during childhood, such as maternal depression.

Indirect support for the differential susceptibility hypothesis comes from two observations. One is that in the study by Thompson *et al*. the differences between high and low scores on psychopathology ratings were larger for the AG genotype; that is, subjects who carried the AG genotype and had no experience of maternal depression had lower scores on depressive symptoms and social anxiety than both the GG group with adversity and the GG group without adversity [63]. By extrapolation, one could assume that subjects carrying the AG genotype would have scored even lower on measures of psychopathology if exposed to thriving environmental conditions during childhood. This conclusion is, at present, to some extent speculative; however, it makes sense in light of a critical remark by Belsky *et al.*, suggesting that treating the absence of adversity as the 'good end' of functioning may lead to an under-detection of differential susceptibility [12].

The other observation indirectly supporting the notion of differential susceptibility pertains to anatomical differences associated with OXTR polymorphic variation. The volume of the amygdala has been found to increase in a 'dose dependent' manner, depending on whether psychologically healthy individuals carry no, one or two A alleles of the rs2254298 SNP [64,65], even though this seems to be influenced by methodological issues, that is, whether one chooses a voxel-based morphometric approach [66] or a manual-tracing methodology [65,67].

With respect to psychopathology, a greater than average volume of the amygdala has been found in children with autism [69]. In light of the association of the A allele with autism, at least in Asian populations [55,56], one could therefore conclude that the A is 'just' a risk allele, hence conferring increased vulnerability for psychiatric disorder. When looking at adults, however, a different picture emerges. In adults with autism, the amygdala size is reduced, rather than increased [70]. Moreover, in psychologically healthy adults, the amygdala volume is correlated with social network size [68]. This speaks against a simple relationship of amygdala size with autism that is mediated by the OXTR genotype. Instead, it is conceivable that the A load at the rs2254298 SNP of *OXTR*increases one's susceptibility to environmental conditions in ways that can either produce an autistic phenotype if associated with adverse environmental conditions, yet can also lead to greater sociability (in other words, the opposite phenotype in the same domain), if adversity is absent or environmental conditions beneficial. Notably, environmental adversity does not necessarily mean that unfavourable effects on development occur postnatally. It could well be the case that adverse environmental contingencies strike much earlier. For example, in the case of autism, Ronald *et al*. [71] reported that prenatal maternal stress has a small, but significant effect on the development of autistic traits in two-year-old boys (reviewed in [72]).

From an evolutionary point of view, it is relevant in the context of differential susceptibility that the A allele of rs2254298 emerged as a mutation at some point during human evolution, while the G allele is the ancestral variant [54]. In addition, there are profound differences in the frequency of A alleles between human populations. The question whether or not these population differences are due to founder effects, positive selection or a selective sweep is unclear. In light of the putative association of rs2254298A with amygdala size [64,65], and the link of amygdala volume with social complexity [68], one can at least not exclude positive selection at some point during human evolution, provided that a larger social network size has paid-off reproductively, which is in agreement with the social brain hypothesis [16,17]. Consistent with this interpretation, it is conceivable that the A allele of the rs2254298 SNP of *OXTR *is similarly linked with developmental plasticity as, for example, polymorphisms of the DRD4 [7,8], where ethnic differences in frequency are equally large.

The present article yields several limitations. One is that the functionality of the SNP of *OXTR *is unknown, all the more as it is located in a non-coding region of *OXTR*. Nor is anything known about how polymorphic variation of the OXTR or the interaction between genetic variation and environment (epigenetic effects) translate into differential availability of OXT in the brain, an issue that may account for the diverse findings regarding plasma or CSF levels of OXT in psychopathological conditions. Moreover, genetic variation at a single locus cannot be viewed upon in isolation, since epistasis (interaction between two or more genes) is prevalent. For example, with regard to the differential susceptibility hypothesis, Belsky and Beaver [73] recently reported that the individual load of such alleles can exert additive effects on behaviour. They examined five loci, namely the DRD2, DRD4, 5-HTTLPR, dopamine transporter and MAO-A, in adolescents and found that the more susceptibility alleles one possessed, the greater an individual's self-regulation abilities hinged upon early environmental contingencies, such as the emotional bond to one's mother, for better or for worse. In other words, the fewer susceptibility alleles a person was endowed with, the less dependent self-regulation was on individual genetics. Interestingly, this was statistically significant only in the male participants, which could suggest that men are perhaps more susceptible than women to environmental influences as far as their self-regulatory capacity is concerned [73].

Another important issue is that the interpretation of the findings pertaining to rs2254298 cannot simply be generalized to other SNPs of *OXTR*. For example, the SNP rs53576 of *OXTR*, which is also located in the third intron and bears a G→A mutation, has been associated with increased stress reactivity and lower empathy scores in psychologically healthy patients of mixed ethnicity carrying at least one A allele [74]. In contrast, another study found that male GG carriers showed the highest sympathetic cardiac reactivity to psychological stress compared to A carriers (no such difference emerged in women) [75], yet in another study displayed lower cortisol responses to stress when receiving social support from their partner or close friend than A carriers [76]. Moreover, individuals with a GG or GA genotype were more likely to seek emotional support when distressed than AA carriers. However, this was evident only in North Americans, but not Koreans where social support seeking is less common, suggesting that culture modulates the interaction of stress-related behaviour with genetic factors [77]. No conclusive evidence has emerged with regard to attachment style (as a potential clue to the quality of early experiences) and rs53576 [24]. However, it has been reported that the SNP rs53576 of *OXTR *makes an independent contribution to lower levels of parental responsiveness to the needs of young children, similar to the effects of the s-allele of the HTTLPR, in a large sample of Caucasian mothers [78]. That is, the presence of at least one A allele of rs53576 explained 3% of the variance of maternal sensitivity, with the same amount being accounted for by the short serotonin transporter allele, yet no interaction or potentiating effect of the two SNPs was found. Taken together, in this case the A allele of rs53576 seems to confer greater vulnerability to unfavourable psychological outcome, yet it is unclear if this is compensated, and what the mechanism could be that accounts for the observation that the A allele is not selected against (but, instead, maintained at high frequencies in some populations).

In any event, given the importance of OXT for sexual behaviour, parturition and lactation in mammals, such compensatory effects could well reside outside the nervous system. However, surprisingly little research has examined the impact of variation of the OXTR on reproduction. One study suggests that OXT influences the micro-environment surrounding the developing oocyte and is therefore involved in regulating fertility and embryo development [79]. Even more interestingly, another study involving over 1,000 male and 1,000 female participants reported that polymorphic variation of the OXTR impacted the reproductive behaviour of women. Specifically, Caucasian women who carried the long version of the OXTR used contraceptives less and had children at an earlier age compared to women possessing the short variant (whereas male sexual behaviour was influenced by variation of the arginine-vasopressin receptor gene). Put another way, women's reproductive behaviour and life history traits were influenced by variation of the OXTR [80]. Unfortunately, as yet no information is available as regards rs2254298 on sexual behaviour and reproduction.

## Conclusions

Polymorphic variation at the OXTR is a candidate for differential susceptibility to environmental contingencies, which, if associated with adversity during early childhood or even the prenatal period, may result in a greater risk of developing psychopathologies such as autism. Conversely, under thriving conditions the same variation may have advantageous effects on an individual's social network, hence supporting the view that all genetic variation may not unequivocally be linked to greater vulnerability but, rather, to increased plasticity. With respect to variations at *OXTR*, gene-environment interactions are poorly studied, such that more information is warranted, especially concerning the effects of positive environmental conditions (for example, emotional warmth and acceptance) on neurodevelopment, not just the absence of adversity [12]. Moreover, to date it is unclear how exactly polymorphic variation at the OXTR translates into the actual availability of OXT in the brain. Similarly, it is poorly understood how variation of the OXTR interacts with cultural differences and differences between male and female psychology. Finally, examining the physiological effects of OXTR polymorphisms on sexuality and reproductive behaviour could broaden the picture of the functionality of the oxytocinergic system.

In summary, this overview is one of the first that has argued for an association of genetic plasticity with both behavioural and neuroanatomical correlates of gene-environment interaction. Even though more studies are needed to clarify several open questions, the diathesis-stress model of psychopathological conditions needs to be refined by anchoring clinical research on gene-environment interaction in evolutionary theory [81]. Such an approach may also be suitable to inform research on gene-environment interaction in psychiatry in general and other medical disciplines.

## Competing interests

The author declares that he has no competing interests.

## Authors' information

MB is Head of the Research Department of Cognitive Neuropsychiatry and Psychiatric Preventive Medicine at the LWL University Hospital of Psychiatry, Ruhr-University Bochum, Germany. One of his main research objectives is to improve understanding of psychiatric disorders by looking at psychopathological conditions through the lens of evolutionary theory.

## Pre-publication history

The pre-publication history for this paper can be accessed here:

http://www.biomedcentral.com/1741-7015/10/38/prepub
